# Association between poor oral health and deterioration of appetite in older age: results from longitudinal analyses of two prospective cohorts from the UK and USA

**DOI:** 10.1136/bmjopen-2024-083973

**Published:** 2025-02-03

**Authors:** Suruchi G Ganbavale, Ziyi Cai, John C Mathers, Olia Papacosta, Lucy Lennon, Peter H Whincup, Robert Weyant, S Goya Wannamethee, Sheena E Ramsay

**Affiliations:** 1 Newcastle University Population Health Sciences Institute, Newcastle Upon Tyne, UK; 2 Department of Public Health, Policy and Systems, University of Liverpool Institute of Population Health, Liverpool, UK; 3 Human Nutrition Research Centre, Newcastle University, Newcastle upon Tyne, UK; 4 Department of Primary Care and Population Health, University College London, London, UK; 5 City St George's, University of London, London, UK; 6 Dental Public Health, University of Pittsburgh, Pittsburgh, Pennsylvania, USA; 7 Institute of Health & Society, Newcastle University, Newcastle upon Tyne, UK

**Keywords:** Aging, PUBLIC HEALTH, NUTRITION & DIETETICS, Aged, Health Equity

## Abstract

**Abstract:**

**Objective:**

This study investigated the association of poor oral health with appetite loss and deterioration in appetite longitudinally in older adults.

**Design:**

Cross-sectional and longitudinal observational study.

**Setting:**

Data came from two population-based cohorts of older adults from the UK and USA.

**Participants:**

The British Regional Heart Study (BRHS) included men (n=1348, age=79–87 years in 2016–2017 at baseline and 81–89 years in 2018–2019 at follow-up). The US Health, Aging and Body Composition (HABC) Study included men and women (n=2998, age=71–77 years in 1998–1999 at baseline and 73–79 years in 2000–2001 at follow-up). Objective and self-reported oral health measures were collected.

**Outcome measures:**

Loss of appetite, at baseline and 2-year follow-up, was based on the Simplified Nutrition Assessment Questionnaire in the BRHS and self-reported appetite loss in the HABC Study. In the BRHS, changes in oral health over time were also assessed. Logistic regression models were adjusted for sociodemographic, behavioural and health-related factors.

**Results:**

Cross-sectionally, poor self-rated oral health, dry mouth, eating or chewing difficulty, food avoidance and cumulative oral health problems were associated with appetite loss in both studies. Longitudinally, in the BRHS, dry mouth (OR=2.12 (95% CI=1.40 to 3.20)), eating or chewing difficulty (OR=1.59 (95% CI=1.02 to 2.48)), food avoidance (OR=1.75 (95% CI=1.16 to 2.65)) and cumulative oral health problems (OR=2.84 (95% CI=1.80 to 4.50)) at baseline were associated with sustained poor/deterioration in appetite over the follow-up, after full adjustment. In the HABC Study, self-rated oral health ((OR=1.13 (95% CI=1.01 to 1.27)), tooth loss (OR=1.78 (95% CI=1.15 to 2.76)), dry mouth (OR=1.76 (95% CI=1.02 to 3.03)), eating or chewing difficulty (OR=1.88 (95% CI=1.41 to 2.50)) and cumulative oral health problems (OR=1.89 (95% CI=1.33 to 2.70)) at baseline were associated with sustained poor/deterioration in appetite during follow-up. In the BRHS, sustained poor/deterioration in oral health markers (self-rated oral health, dry mouth, eating or chewing difficulty, food avoidance, loose denture/s) over the follow-up were associated with sustained poor/deterioration of appetite.

**Conclusion:**

Oral health is a potentially important contributor to maintaining good appetite in older age.

**STRENGTHS AND LIMITATIONS OF THIS STUDY**

- This study investigated cross-sectional and prospective associations of a comprehensive set of oral health measures with appetite loss in two population-based cohort studies of older adults in the UK and the USA.
- Additionally, this study examined the association of changes in oral health over time with changes in appetite loss prospectively using UK-based cohort data.
- The UK-based cohort comprised White British men only, and the USA-based cohort included White and African American men and women living in Pittsburgh and Memphis, USA. Consequently, the generalisability of the study findings to women and other ethnic groups needs to be further investigated.
- In the USA-based cohort, dry mouth and appetite loss were assessed using single-item questions, which may not have fully captured the extent of these conditions in this cohort sample.
- This study examined the association between poor oral health and deterioration of appetite in older adults after adjusting for their individual sociodemographic characteristics and health-related conditions and behaviours in both UK and USA-based cohorts. However, the information on their dental history and reasons for tooth loss were not available for either cohort. Consequently, the possibility of residual confounding remains in this study.

## Introduction

Appetite loss in older adults (‘anorexia of ageing’), characterised as decline in appetite and food intake, is a major public health concern.^1^ Between 5% and 25% of community-dwelling older adults in developed countries experience appetite loss.^2^ This prevalence of appetite loss may be as high as 62% in hospitalised populations and 85% in nursing home populations.^2^ Persistent appetite loss in older age can lead to poor quality of life, malnutrition, unintentional weight loss, frailty and sarcopenia, thereby increasing the risk of mortality.^1^,^5^ Understanding contributors to appetite loss is, therefore, important to help maintain optimum appetite in later life. Appetite loss is a multifactorial health condition.^1 3 6 7^ Factors that contribute to appetite loss in older age are pathophysiological factors, including inability to feed oneself, lowered metabolic rate, reduced physical activity, sensory impairments (taste, smell and vision), increased activity of satiety factors (eg, cholecystokinin); chronic diseases, including pulmonary disease, abdominal ischaemia, cancer, recurrent infections; and psychosocial factors including, poverty, difficulty in shopping and preparing food, lack of socialisation, loneliness and depression; and neurocognitive factors, including dementia.^1 3 6 7^

Alongside these factors, poor oral health is recognised as a potential contributor to loss of appetite in older age.^7^,^9^ A cross-sectional study in Japan^10^ revealed that poor oral health status (based on teeth and other oral tissues (eg, gums), saliva, dentures, oral hygiene and dental pain) was associated with appetite loss in older adults aged ≥65 years. Another study, from Australia, reported that men aged ≥76 years with poor appetite were more likely to wear dentures, had limited chewing capacity, dry mouth and poorer self-evaluated oral health.^11^ Most of the current evidence^7^,^11^ is from cross-sectional studies and the prospective relationship between poor oral health and appetite loss remains underexplored in ageing populations.

Therefore, we investigated the association of poor oral health with appetite loss and deterioration in appetite over time in older adults. We employed a range of oral health measures, both subjective and objective measures, and examined their associations longitudinally with appetite loss, and with the changes in appetite over time, using two cohort studies of community-dwelling older adults, in the UK and the USA. Additionally, we investigated the association of changes in oral health measures with changes in appetite over time.

## Methods

Data for this cross-sectional and longitudinal observational study came from two population-based cohorts of older adults from the UK and the USA described below.

### The British Regional Heart Study (BRHS)

The British Regional Heart Study (BRHS), an ongoing cohort study that commenced in 1978–1980, comprises a socioeconomically and geographically representative sample of 7735 men (aged 40–59 years) registered with general practices across 24 towns in Great Britain.^12^ In 2016–2017, surviving participants (aged 76–96 years, n=2076) were invited to complete a postal questionnaire, including self-reported medical history, general health, and oral health, appetite, socioeconomic conditions, and physical function (n=1348, response rate=65%). These data served as the baseline in the present study. In 2018–2019, surviving participants (aged 78–98 years, n=1633) underwent a physical examination, involving an objective oral examination undertaken by a dental hygienist (n=667, response rate=41%), and completed a similar questionnaire as of 2016–2017 (n=1009, response rate=62%), serving as a follow-up in the present study. Details on assessments and validity have been reported.^13 14^ Ethical approval was granted by the National Research Ethics Service (NRES) Committee, London Central region. Participants provided written informed consent in accordance with the Declaration of Helsinki.

### The Health, Aging, and Body Composition (HABC) Study

The Health, Aging and Body Composition (HABC) Study commenced in 1997–1998^15^ and included a socially representative cohort (ie, representing different education attainment levels) of 3075 White and African American men and women aged 68–79, able to walk a quarter of a mile or climb 10 steps without a difficulty at the time of initial examination. Participants lived across two sites that is, Memphis, Tennessee and Pittsburgh, PA in the USA. Individuals of White ethnicity were sampled from Medicare enrollees whereas African American participants were sampled from designated zip codes within the study sites.

In 1998–1999 (year 2 of the HABC Study), a total of 2998 of 3040 (response rate=99%) surviving participants aged 69–80 years underwent physical assessments including health status, symptoms, behaviours, medications, psychosocial conditions, anthropometric measurements and physical functioning, provided blood samples, and completed questionnaires involving questions on oral health and appetite. Data were collected via clinic or home visits, which aided in a high response rate. Additionally, 1975 participants attended an objective oral examination undertaken by dental hygienists or periodontists. Year 2 data served as the baseline in the present study. In 2000–2001 (year 4 of the HABC Study), a total of 2700 of 2702 surviving participants were again followed-up through a physical assessment and questionnaire survey, serving as a follow-up in the present study. Ethical approval was granted by the University of Pittsburgh, the University of Tennessee, Memphis, the University of California San Francisco, and the US National Institutes of Health. Written informed consent was gained from participants.

### Measures of oral health

In the BRHS, the number of natural teeth was measured using a single question (ie, Do you have any of your natural teeth?: yes/no) at baseline, and using the same question and a count of natural teeth (which can range from 0 to 32 teeth) as part of the oral examination at follow-up. In the HABC Study, participants had an oral examination, which included a count of natural teeth. Both studies included self-reported measures on self-rated oral health (excellent, good, fair or poor),^12 14 15^ dry mouth (measured using the Xerostomia Inventory Scale^16^ in the BRHS, and a single question (Does your mouth feel dry when eating?) in the HABC Study), difficulty eating or chewing foods due to dental problems (ie, problems related to mouth, teeth or denture/s), food avoidance due to dental problems, and denture/s use, which were grouped as yes/no. Online supplemental appendix 1 table 1 presents the key oral health measures available at baseline and follow-up in both cohorts.

### Measures of changes in oral health

The BRHS cohort had information on oral health at baseline and follow-up, including tooth loss (edentulism), self-rated oral health (excellent, good, fair, poor), number of dry mouth problems, difficulty eating or chewing foods due to dental problems (some/no difficulty), food avoidance due to dental problems (yes/no), loose denture/s (yes/no) and number of cumulative oral health problems. Changes in these oral health markers over the follow-up period were examined. These measures/variables were categorised as sustained good/improved and sustained poor/deterioration in oral health over time. Detailed description of sustained poor/deterioration in each oral health measure is provided in online supplemental appendix 2 figure 1. Since the HABC cohort was not followed up for oral health measures over time, it was not possible to calculate changes in oral health among participants of the HABC Study.

### Measures of appetite loss

In the BRHS, appetite loss was measured using the Simplified Nutritional Appetite Questionnaire (SNAQ) at baseline and follow-up.^17^ SNAQ is a validated, self-reported measure for assessing appetite in older adults.^8 10^ It comprises four questions in total with their responses ranging from 1=very poor to 5=very good. SNAQ score of ≥14 to 20 indicates appetite loss with a significant risk of ≥5% weight loss within 6 months.^17^ BRHS participants with an SNAQ score of ≥14 were categorised as having loss of appetite.^8 10^ Information on SNAQ score collected at baseline was used in to examine the cross-sectional relationships between measures of poor oral health and appetite loss. Information on SNAQ score collected at follow-up was used to determine changes in appetite loss over time.

In the HABC Study, appetite loss at baseline was determined through a self-reported question during a clinic visit (ie, In the past month, would you say that your appetite or desire to eat has been?). Participants not attending the clinic visit were asked a similar question during the home visit (ie, In general, would you say that your appetite or desire to eat has been?). Responses ranged from 1=very good to 5=very poor. Participants reporting having moderate/poor/very poor appetite were categorised as having appetite loss at baseline.^7^ This question was repeated at the follow-up, which was used to determine changes in appetite loss over time.

In both studies, changes in appetite loss were categorised as sustained good/improved (no appetite loss at baseline and at follow-up; or appetite loss at baseline but not at follow-up) and sustained poor/deteriorated (appetite loss at baseline and at follow-up; or no appetite loss at baseline but reported appetite loss at follow-up).

### Covariates

In the BRHS, covariates included age, social class, smoking status, alcohol consumption, and history of diabetes or cardiovascular disease (CVD), and dry mouth medications. Age was a continuous variable. For social class, occupational categories including I-professionals, II-managerial and III-semiskilled non-manual were grouped as non-manual; and III-semiskilled manual, IV-partly skilled and V-unskilled were grouped as manual (based on the Registrar General’s Social Class classification).^13 14 18^ Participants were categorised as current smokers, former smokers (gave up before 2016–2017) and those who never smoked.^19^ Alcohol intake was dichotomised as moderate to heavy drinkers (ie, having 5–6 drinks or ˃6 drinks daily or on most days of the week), and occasional or non-drinkers.^20 21^ History of diabetes was assessed via self-reported history of diagnosis. History of CVD included self-reported doctor-diagnoses of angina, heart attack or heart failure. Dry mouth medications were based on participants taking medications with dry mouth as a side effect and were grouped into 0, 1 and ≥2 medications.

In the HABC Study, age, gender, race, education level, smoking status, history of diabetes or CVD were employed as covariates. History of CVD included self-reported doctor-diagnosis of heart attack, angina, stroke or heart failure. Age was a continuous variable; gender was dichotomised as male and female; race included White, American African and other; education level (high school or lower, high school graduate, post-high school)^20^; smoking status was categorised as current smoker, former smoker and having never smoked.^20^ Alcohol consumption was not measured at baseline in the HABC Study.

### Statistical analyses

Descriptive analyses were undertaken to assess the key characteristics of both cohorts, where mean and standard deviation (SD) were calculated for continuous variables and the percentages were calculated for categorical variables.

For oral health measures, tooth loss was categorised as with or without any natural teeth (ie, complete tooth loss) in the BRHS and the HABC Study. Self-rated oral health was categorised as excellent/good vs fair/poor;^19^ dry mouth was grouped as 0, 1–2 or ˃2 symptoms (BRHS),^19^ or as yes/no (HABC). All other oral health variables were categorised as yes/no.

Additionally, a cumulative measure of oral health was computed to quantify the number of oral health problems experienced by each participant in both cohorts to assess the severity of poor oral health.^20^ Oral health measures included complete tooth loss (edentulism); dry mouth; difficulty eating or chewing foods; food avoidance due to dental problems; sensitivity to hot, cold or sweet foods (BRHS); and loose dentures (BRHS). Cumulative oral health problems were categorised as 0, 1, or ≥2 oral health problems.^19^

Logistic regression was undertaken to examine cross-sectional relationships between each oral health marker and appetite loss, separately using the baseline data from both studies, to obtain ORs and 95% CIs. Models were first adjusted for age, and then further adjusted for other covariates.

In the BRHS, prospective assessments included (i) the associations of oral health measures at baseline with changes in appetite loss from baseline to follow-up and (ii) the associations of changes in oral health measures with changes in appetite loss over the follow-up period. Logistic regression models were performed to obtain ORs (95% CIs) for sustained poor/deterioration in appetite according to measures of oral health and changes in oral health, respectively. Models were first adjusted for age, and then further adjusted for other covariates.

In the HABC Study, similar prospective analyses, involving logistic regressions, were undertaken to examine the associations of oral health measures at baseline with changes in appetite loss from baseline to follow-up. Age-adjusted and fully adjusted models were employed to obtain ORs (95% CIs) for poor/deterioration in appetite according to oral health measures.

All analyses were undertaken separately for each cohort using SAS 9.4. The study adhered to STROBE (Strengthening the Reporting of Observational Studies in Epidemiology) guidelines (https://www.strobe-statement.org/).

## Results

Table 1 presents baseline characteristics of the BRHS (n=1348) and HABC (n=2998) cohorts. The mean age of the BRHS and the HABC samples was 83 years (SD=4 years) and 74 years (SD=3 years), respectively. The BRHS included men. In the HABC Study, 52% participants were male and 40% participants were African American. In the BRHS, 45% participants were from manual occupational social class. In the HABC Study, 24% participants did not attain high school graduation. Three per cent and nine per cent of participants were current smokers in the BRHS and HABC Study, respectively. In the BRHS, 28% of the participants had a history of CVD, which was 3% in the HABC participants. Eighteen per cent and five per cent of the participants had a history of diabetes in the BRHS and HABC Study, respectively. In the BRHS, 38% and 23% of the participants had at least 1 and ≥2 cumulative oral health problems, respectively. Twenty-three per cent of the participants in the BRHS experienced appetite loss at baseline and 24% of the participants experienced sustained poor/deteriorated appetite from baseline to follow-up. In the HABC Study, 19% and 12% of the participants had at least 1 and ≥2 cumulative oral health problems, respectively. Nineteen per cent of the HABC participants experienced appetite loss at baseline and 12% of the participants experienced sustained poor/deterioration of appetite from baseline to follow-up.

**Table 1:** Baseline characteristics of participants in the BRHS (2016–2017) and Health ABC Study (1998–1999)

Baseline characteristics	BRHS (n=1348)	Health ABC Study (n=2998)
Age mean (SD)	83 (4)	74 (3)
Gender: male	1348 (100%)	1575 (52%)
Race		
White	1036 (76.8%)	1628 (60%)
Other*	4 (0.3%)	1072 (40%)
Manual occupational social class	587 (45%)	Not assessed
Education level attained		
High school or lower	247 (18.3%)	644 (24%)
High school graduate	–	874 (32%)
Post-high school	988 (73.3%)	1161 (43%)
Data missing	113 (8.3%)	21 (0.8%)
Smoking status		
Never smoked	519 (38%)	1213 (45%)
Current smokers	38 (3%)	251 (9%)
Former smokers	788 (58%)	1218 (45%)
History of cardiovascular disease	377 (28%)	92 (3%)
History of diabetes	242 (18%)	129 (5%)
Moderate or heavy alcohol consumption	47 (4%)	Not assessed

* Note: In the HABC Study, the category ‘Other’ for the variable ‘Race’ was entirely comprised of African American adult population.

BRHSBritish Regional Heart Study

Table 2 presents the prevalence of oral health measures, and ORs (95% CIs) for appetite loss according to measures of oral health from cross-sectional analyses undertaken using baseline data from the BRHS and the HABC Study. In the BRHS, age-adjusted models demonstrated that the odds of appetite loss were higher for those reporting fair/poor self-rated oral health (OR=1.83 (95% CI=1.39 to 2.42)) compared with those reporting excellent/good oral health; and this remained significant in fully adjusted models. The risk of appetite loss was higher for those with complete tooth loss (OR=1.84 (95% CI=1.34 to 2.55)) on full adjustment. Participants having ˃2 symptoms of dry mouth had an increased risk of experiencing appetite loss (fully adjusted OR=2.84 (95% CI=1.95 to 4.15)). Increased risk of appetite loss was also observed for those having difficulty eating or chewing foods, avoiding foods, those using denture/s and those who had loose denture/s (fully adjusted OR=1.86 (95% CI=1.24 to 2.81)). Teeth sensitivity, which was available in the BRHS cohort, was not significantly associated with appetite loss in the age-adjusted model (OR=1.23 (95% CI=0.77 to 1.95)). In the HABC Study, while tooth loss was associated with appetite loss in age-adjusted model, this was not significant on full adjustment. The risk of appetite loss was greater in participants with poor self-rated oral health, dry mouth, having difficulty eating or chewing foods, avoiding foods, and those with having ≥2 oral health problems in both age-adjusted and fully adjusted models.

**Table 2:** Cross-sectional associations between oral health measures and loss of appetite in the BRHS (2016–2017) and Health ABC Study (1998–1999)

Oral health markers	BRHS data (n=1348)			HABC Study data (n=2998)		
	Number of participants(n=1348)	Model A*	Model B†	Number of participants(n=2998)	Model C‡	Model D§
		OR (95% CI)	OR (95% CI)		OR (95% CI)	OR (95% CI)
Self-rated oral health	**1301**			**2718**		
Excellent/good	n=884 (68%)	1.00	1.00	n=1889 (70%)	1.00	1.00
Fair/poor	n=417 (32%)	**1.83(1.39to2.42)**	**1.80(1.35to2.40)**	n=829 (30%)	**1.28(1.16to1.40)**	**1.22(1.11to1.35)**
Tooth loss	**1323**			**1973**		
Have natural teeth	n=1054 (80%)	1.00	1.00	n=1765 (90%)	1.00	1.00
No natural teeth	n=269 (20%)	**2.12(1.57to2.86)**	**1.84(1.34to2.55)**	n=208 (10%)	**1.49(1.05to2.13)**	1.42 (0.97 to 2.08)
Dry mouth	**1343**					
0 problems	n=367 (27%)	1.00	1.00	–	–	–
1-2 problems	n=405 (30%)	1.16 (0.77 to 1.73)	1.18 (0.77 to 1.81)	–	–	–
>2 problems	n=571 (43%)	**2.83(2.00to4.01)**	**2.84(1.95to4.15)**	–	–	–
**Dry mouth score (per unit increase in number of symptoms)**		**1.20(1.15to1.26)**	**1.19(1.14to1.25)**	–	–	–
Dry mouth				**2719**		
Does not experience dry mouth		–	–	n=2612 (96%)	1.00	1.00
Experiences dry mouth		–	–	n=107 (4%)	**2.18(1.403to3.38)**	**2.03(1.29to3.18)**
Difficulty eating or chewing foods	**1340**			**2725**		
No difficulty	n=1155 (86%)	1.00	1.00	n=2191 (80%)	1.00	1.00
Some difficulty	n=185 (14%)	**2.66(1.91to3.71)**	**2.26(1.59to3.22)**	n=534 (20%)	**1.72(1.37to2.17)**	**1.60(1.26to2.04)**
Food avoidance due to dental problems	**1326**			**2722**		
No	n=1095 (83%)	1.00	1.00	n=2334 (86%)	1.00	1.00
Yes	n=231 (17%)	**3.14(2.30to4.28)**	**2.61(1.88to3.62)**	n=388 (14%)	**1.95(1.51to2.51)**	**1.78(1.37to2.32)**
Denture/s use	**1336**			**2757**		
Does not wear denture/s	n=597 (45%)	1.00	1.00	n=1867 (68%)	1.00	1.00
Wears denture/s	n=739 (55%)	**1.41(1.08to1.85)**	**1.37(1.03to1.82)**	n=890 (32%)	1.09 (0.88 to 1.34)	1.02 (0.82 to 1.27)
Cumulative oral health problems	**1346**			**2759**		
0 problems	n=521 (39%)	1.00	1.00	n=1910 (69%)	1.00	1.00
1 problem	n=517 (38%)	**1.82(1.31to2.52)**	**1.84(1.31to2.57)**	n=529 (19%)	**1.28(1.00to1.65)**	1.27 (0.97 to 1.64)
≥2 problems	n=308 (23%)	**4.70(3.29to6.70)**	**4.20(2.88to6.14)**	n=320 (12%)	**2.40(1.82to3.16)**	**2.14(1.60to2.85)**

OR (95% CI) written in bold indicate p<0.05.

* Adjusted for age.

† Adjusted for age, individual social class, smoking status, alcohol consumption, history of diabetes or CVD.

‡ Adjusted for age.

§ Adjusted for age, gender, race, education, smoking status, history of diabetes or CVD.

BRHSBritish Regional Heart StudyHABCHealth, Aging and Body Composition

Table 3 presents OR (95% CI) for prospective associations of oral health measures with changes in appetite loss over time in the BRHS. Participants with ˃2 dry mouth symptoms had a greater risk of poor/deterioration in appetite (age-adjusted OR=2.30 (95% CI=1.56 to 3.38)) compared with those without dry mouth symptoms, and this remained significant on full adjustment. Participants having difficulty eating or chewing foods (OR=1.59 (95% CI=1.02 to 2.48)) and food avoidance (OR=1.75 (95% CI=1.16 to 2.65)) due to dental problems had greater risks of poor/deterioration in appetite on full adjustment. In the age-adjusted model, the risk of poor/deterioration in appetite increased in participants using dentures (OR=1.43 (95% CI=1.04 to 1.96)); however, this association became statistically insignificant on full adjustment. Cumulatively, participants having one oral health problem (fully adjusted OR=1.61 (95% CI=1.11 to 2.34)) had higher risk of poor/deterioration in appetite. This risk increased even further in participants with ≥2 oral health problems (fully adjusted OR=2.84 (95% CI=1.80 to 4.50)).

**Table 3:** Longitudinal associations between oral health measures at baseline (2016–2017) and deterioration of appetite loss (measured using the SNAQ score) over a 2-year follow-up in the BRHS

Oral health markers	Number of participants (n=934)	Model A*	Model B†
		OR (95% CI)	OR (95% CI)
Self-rated oral health	**912**		
Excellent/good	653	1.00	1.00
Fair/poor	259	1.22 (0.87 to 1.73)	1.18 (0.82 to 1.68)
Tooth loss	**921**		
Has natural teeth	761	1.00	1.00
No natural teeth	160	1.35 (0.91 to 2.01)	1.16 (0.77 to 1.77)
Dry mouth	**932**		
0 problems	265	1.00	1.00
1–2 problems	285	0.88 (0.56 to 1.38)	0.84 (0.52 to 1.36)
˃2 problems	382	**2.30(1.56to3.38)**	**2.12(1.40to3.20)**
**Dry mouth score (per unit increase in number of symptoms)**	**932**	**1.16(1.09to1.23)**	**1.14(1.07to1.22)**
Difficulty in eating or chewing foods	**931**		
No difficulty	815	1.00	1.00
Some difficulty	116	**1.89(1.24to2.88)**	**1.59(1.02to2.48)**
Food avoidance due to dental problems	**923**		
Yes	781	1.00	1.00
No	142	**1.99(1.34to2.95)**	**1.75(1.16to2.65)**
Denture/s use	**926**		
Does not use denture	427	1.00	1.00
Uses dentures	499	**1.43(1.04to1.96)**	1.35 (0.97 to 1.88)
Loose denture/s	**499**		
Wears dentures but does not have loose dentures	410	1.00	1.00
Wears dentures and has loose dentures	89	1.60 (0.96 to 2.65)	1.38 (0.81 to 2.36)
Teeth sensitivity	**882**		
No sensitivity	799	1.00	1.00
Experiences sensitivity	83	1.26 (0.75 to 2.12)	1.28 (0.75 to 2.21)
Cumulative oral health problems	**934**		
0 problems	386	1.00	1.00
1 problem	395	**1.75(1.22to2.51)**	**1.61(1.11to2.34)**
≥2 problems	153	**3.09(2.01to4.77)**	**2.84(1.80to4.50)**

OR (95% CI) written in bold indicate p<0.05.

* Model A: Adjusted for age Adjusted for age, individual social class, smoking status, alcohol consumption, history of diabetes or CVD.

† Adjusted for age, individual social class, smoking status, alcohol consumption, history of diabetes or CVD.

BRHSBritish Regional Heart StudySNAQSimplified Nutritional Appetite Questionnaire

Table 4 presents the results from prospective analyses between oral health measures at baseline and changes in appetite loss from baseline to follow-up in the HABC Study. In the fully adjusted models, the risk of having sustained poor/deterioration in appetite was higher among the participants having fair/poor self-rated oral health (OR=1.13 (95% CI=1.01 to 1.27)), those with no natural teeth, dryness of mouth, difficulty eating or chewing foods, and with cumulative oral health problems than their counterparts.

**Table 4:** Longitudinal associations between oral health measures at baseline (1998–1999) and deterioration of appetite loss (measured using the self-rated appetite variable) over a 2-year follow-up in the Health ABC Study

Oral health markers	Number of participants (n=2700)	Model A*	Model B^†^
		OR (95% CI)	OR (95% CI)
Self-rated oral health	**2488**		
Excellent/good	1754	1.00	1.00
Fair/poor	734	**1.23(1.10to1.38)**	**1.13(1.01to1.27)**
Tooth loss	**1830**		
Has natural teeth	1642	1.00	1.00
No natural teeth	188	**2.14(1.43to3.22)**	**1.78(1.15to2.76)**
Dry mouth	**2490**		
Yes	2393	1.00	1.00
No	97	**1.93(1.13to3.30)**	**1.76(1.02to3.03)**
Difficulty in eating or chewing foods	**2494**		
No difficulty	2021	1.00	1.00
Some difficulty	473	**2.07(1.57to2.74)**	**1.88(1.41to2.50)**
Food avoidance due to dental problems	**2492**		
Yes	2152	1.00	1.00
No	340	1.31 (0.94 to 1.84)	1.09 (0.77 to 1.55)
Denture/s use	**2521**		
Does not wear denture/s	1706	1.00	1.00
Wears denture/s	815	1.19 (0.92 to 1.54)	1.08 (0.83 to 1.41)
Cumulative oral health problems	**2522**		
0 problems	1763	1.00	1.00
1 problem	479	**1.55(1.15to2.11)**	**1.48(1.08to2.02)**
≥2 problems	280	**2.35(1.67to3.30)**	**1.89(1.33to2.70)**

OR (95% CI) written in bold indicate p<0.05.

* Model A: Adjusted for age Adjusted for age, gender, race, education, smoking status, history of diabetes or CVD.

† Adjusted for age, gender, race, education, smoking status, history of diabetes or CVD.

Table 5 presents ORs (95% CI) for prospective relationships between changes in oral health measures and changes in appetite loss over time in the BRHS. Sustained poor/deterioration in self-rated oral health compared with those reporting sustained good/improved self-rated oral health was associated with a higher risk of sustained poor/deterioration in appetite (OR=1.59 (95% CI=1.12 to 2.24)). Sustained poor/deterioration in dentition was not associated with sustained poor/deterioration in appetite. Having sustained poor/deterioration in dry mouth was associated with sustained poor/deterioration in appetite on full adjustment (OR=1.84 (95% CI=1.31 to 2.57)). Similarly, increased risks of sustained poor/deterioration in appetite were observed for sustained poor/deterioration in difficulty eating or chewing foods, food avoidance and cumulative oral health problems.

**Table 5:** Longitudinal associations between changes in oral health and deterioration of appetite loss (measured using the SNAQ score) between baseline (2016–2017) and follow-up (2018–2019) in the BRHS

Changes in oral health measures	N=934	Model A*	Model B^†^
		OR (95% CI)	OR (95% CI)
Self-rated oral health	**872**		
Sustained good/improved	574	1.00	1.00
Sustained poor/deteriorated	298	**1.78(1.28to2.46)**	**1.59(1.12to2.24)**
Tooth loss	**842**		
Sustained good	704	1.00	1.00
Sustained poor/deteriorated	138	1.37 (0.90 to 2.08)	1.20 (0.76 to 1.87)
Dry mouth	**910**		
Sustained good/improved	593	1.00	1.00
Sustained poor/deteriorated	317	**1.96(1.43to2.69)**	**1.84(1.31to2.57)**
Difficulty in eating or chewing foods	909		
Sustained good/improved	727	1.00	1.00
Sustained poor/deteriorated	182	**2.64(1.85to3.77)**	**2.44(1.68to3.53)**
Food avoidance due to dental problems	**832**		
Sustained good/improved	676	1.00	1.00
Sustained poor/deteriorated	156	**2.46(1.68to3.60)**	**2.35(1.58to3.50)**
Loose denture/s	**471**		
Sustained good/improved	388	1.00	1.00
Sustained poor/deteriorated	83	**1.79(1.07to2.99)**	**1.78(1.01to3.12)**
Cumulative oral health problems	**929**		
Sustained good/improved	758	1.00	1.00
Sustained poor/deteriorated	171	**2.53(1.76to3.63)**	**2.33(1.60to3.41)**

OR (95% CI) written in bold indicate p<0.05.

* Model A: Adjusted for age Adjusted for age, individual social class, smoking status, alcohol consumption, history of diabetes or CVD.

† Adjusted for age, individual social class, smoking status, alcohol consumption, history of diabetes or CVD.

BRHSBritish Regional Heart StudySNAQSimplified Nutritional Appetite Questionnaire

## Discussion

This study of two cohorts of older adults from the UK and the USA demonstrates that poor oral health is associated with increased risk of appetite loss in later life. Cross-sectional findings showed that self-rated oral health, dry mouth, eating or chewing difficulty, food avoidance, and cumulative oral health problems were associated with appetite loss, independent of other covariates in both cohorts. Notably, we observed that several oral health markers including tooth loss, eating or chewing difficulty, and dry mouth were associated with appetite loss over time. Furthermore, we observed that deterioration in oral health markers (including self-rated oral health, dry mouth, eating or chewing difficulty, food avoidance, loose denture/s, and cumulative oral health problems) over time was associated with deterioration of appetite loss during follow-up in the UK cohort.

The study findings are consistent with those of Nomoto *et al*,^10^ which demonstrated associations between appetite loss and a cumulative measure of oral health status through examination of oral cavity including teeth, gums and dentures; and Kimura *et al*,^8^ which reported associations between poor chewing ability and appetite loss. The relationships of appetite loss with denture/s use, chewing capacity, dry mouth, and self-evaluated oral health were also observed in Takehara *et al*.^11^

Poor oral health, particularly, tooth loss, higher likelihood of wearing denture/s, dry mouth, cumulative oral health problems, difficulty in eating or chewing foods due to dental problems can limit chewing (masticatory) function and intake of certain foods in older adults, thereby reducing their nutritional intake.^22 23^ Tooth loss resulting in higher likelihood of wearing dentures may in turn affect the ability to eat different types of foods and influence the food preferences of older adults.^7^ Dry mouth due to decreased saliva production and side effects of multiple medications taken in older age can also cause difficulty in swallowing foods thereby, impacting the appetite.^24 25^ Furthermore, with increased frailty and reduction in sense of taste and smell, older adults who have poor oral health may have less pleasure in eating food.^26^ Together, these issues of difficulty chewing and swallowing food, poor nutrition, and reduced eating pleasure may result in worsening of appetite over time.

A key strength of this study is that we investigated cross-sectional and prospective associations of a comprehensive set of oral health measures with appetite loss in two large cohorts of older adults from the UK and the USA. Detailed information on sociodemographic, behavioural and health-related factors within these cohorts allowed adjustment of key confounders in the investigations. Furthermore, to the best of our knowledge, this is the first study to examine the association of changes in oral health over time with changes in appetite loss prospectively. The study also has limitations. The BRHS sample comprised White British men only; whereas, the HABC Study included White and African American men and women living in Pittsburgh and Memphis, USA. These inclusion criteria limit the generalisability of our findings to women and other ethnic groups. However, participants in both studies were socially representative and population-based samples. In the HABC Study sample, all participants were free of disability at baseline and had lower frequency of tooth loss and dry mouth and so may have been healthier than the BRHS cohort. In the HABC Study, dry mouth and appetite loss were assessed using single-item questions, which may not have fully captured the extent of these conditions in this cohort sample. Additionally, since repeated measurements of oral health were unavailable in this cohort, the relationship between changes in oral health and changes in appetite loss could not be examined in the HABC Study. In both studies, most oral health measures were subjective (self-reported), which could result in under-reporting or mis-reporting of these measures. Consequently, future research may benefit from the examination of the relationship between objective oral health measures such as number of natural teeth, bleeding gums, growth of oral bacteria and appetite loss in older age.

Even so, consistent findings in both study populations, the prospective nature of investigations, and the strength of estimates reported provide some indication that oral health could potentially have an influence on appetite loss. However, the possibility of residual confounding remains due to unmeasured confounders such as dental history and reasons for tooth loss, and inadequately capturing cofounders such as socioeconomic factors and behavioural factors and their influence across the lifecourse (including diet, smoking), and comorbidities such as dementia and cognitive decline. It is also possible that the observed associations were more a reflection of overall deterioration in physiological systems and age-related changes which influence decline in oral health and other aspects of health, including sensory impairment, and cognitive and physical decline. Nevertheless, these two unique and relatively comparable cohorts of older adults facilitated investigation of, and showed similar relationships between, poor oral health and appetite loss in older age.

## Conclusion

This study of older adults in the UK and USA shows that poor oral health is associated longitudinally with appetite loss in older age, particularly with dry mouth, difficulty eating or chewing foods, food avoidance, and cumulative oral health problems. Consequently, better oral health can potentially play a key role in maintaining good appetite in older populations and so mitigate appetite loss that is very common in older age. Appetite loss may be prevented or improved through timely maintenance of oral health to prevent oral health conditions and their deterioration. Policy makers should consider addressing barriers to maintaining good oral health, such as access to dental services and oral health promotion in older adults, thereby facilitating maintaining adequate appetite in older age and helping to avoid poor quality of life, malnutrition, unintentional weight loss, frailty, sarcopenia and premature mortality. The findings have implications for policy at different levels, including early prevention across the lifecourse, as well as adequate access to dental services, addressing barriers to maintaining good oral health and promoting oral health in older age, and including opportunistic dental health promotion for older adults embedded in other health screening activities. Further prospective investigations involving individuals from different ethnicities are needed to assess the generalisability of these findings.

## supplementary material

10.1136/bmjopen-2024-083973 online supplemental file 1

## Data Availability

Data may be obtained from a third party and are not publicly available.
