# Supplementary material for: Association between poor oral health and deterioration of appetite in older age: results from longitudinal analyses of two prospective cohorts from the UK and USA
Source: BMJ Open. 2025 Feb 3;15(2):e083973. doi: 10.1136/bmjopen-2024-083973 (PMC11795386; doi:10.1136/bmjopen-2024-083973)
Supplement: online supplemental file 1 [file bmjopen-15-2-s001.docx]

**Appendix 1 Table 1 Availability of the key oral health measures at baseline and follow-up time-points in the BRHS and HABC Study**

| **Sr No.** | **Oral health measures** | **Available at baseline (2016-17) in the BRHS** | **Available at follow-up (2018-19) in the BRHS** | **Available at baseline (1998-99) in the HABC Study** | **Available at follow-up (2000-01) in the HABC Study** |
| --- | --- | --- | --- | --- | --- |
| 1 | Self-rated oral health (Excellent/ good; Fair/poor) | ✓ | ✓ | ✓ | - |
| 2 | Tooth loss (have natural teeth; do not have natural teeth) | ✓ | ✓ | - | - |
| 3 | Number of natural teeth (0 to 32) | - | ✓ | ✓ | - |
| 4 | Food avoidance due to dental problems (Yes;No) | ✓ | ✓ | ✓ | - |
| 5 | Denture use (Does not wear denture/s;  Wears denture/s) | ✓ | ✓ | ✓ | - |
| 6 | Loose dentures (Wears dentures but does not have loose dentures; Wears dentures and has loose dentures) | ✓ | ✓ | - | - |
| 7 | Difficulty in eating due to dental or denture related problems (No difficulty; Some difficulty) | ✓ | ✓ | ✓ | - |
| 8 | Teeth sensitivity (No sensitivity; Experiences sensitivity) | ✓ | ✓ | - | - |
| 9 | Dry mouth (0; 1; or >2 problems) | ✓ | ✓ |  | - |
| 10 | Dry mouth score (0 to 11 problems) | ✓ | ✓ | - | - |
| 11 | Dry mouth (Does not experience dry mouth; Experiences dry mouth) | - | - | ✓ | - |

**Appendix 2 Figure 1 Description of the key categories of measures on changes in oral health measures in the BRHS**
