# Supplementary material for: Association between poor oral health and deterioration of appetite in older age: results from longitudinal analyses of two prospective cohorts from the UK and USA
Source: BMJ Open. 2025 Feb 3;15(2):e083973. doi: 10.1136/bmjopen-2024-083973 (PMC11795386; doi:10.1136/bmjopen-2024-083973)
Supplement: Supplementary file 2 [file bmjopen-2024-083973.reviewer_comments.pdf]

PEER REVIEW HISTORY

BMJ Open publishes all reviews undertaken for accepted manuscripts. Reviewers are asked to complete a checklist review form and are provided with free text boxes to elaborate on their assessment. These free text comments are reproduced below.

ARTICLE DETAILS

Title (Provisional)

The association between poor oral health and deterioration of appetite in older age: results from longitudinal analyses of two prospective cohorts from the UK and USA

Authors

Ganbavale, Suruchi; Cai, Ziyi; Mathers, John C; Papacosta, Olia; Lennon, Lucy; Whincup, Peter; Weyant, Robert; Wannamethee, Goya; Ramsay, Sheena E

VERSION 1 - REVIEW

Reviewer 1
Name Hakeem, Faisal
Affiliation King's College London, Faculty of Dentistry, Oral & Craniofacial Sciences
Date 07-Mar-2024
COI None

This study presents a compelling analysis of the cross-sectional and longitudinal relationship between oral health and appetite loss among two cohorts of older adults. The research contributes valuable insights to the existing literature. Despite several limitations, the authors have adeptly addressed these in the discussion section. Below are a few minor suggestions aimed at improving the manuscript's readability.

Abstract:

Please specify the age range for clarity: "The British Regional Heart Study (BRHS) encompassed male 0 (n=1348, aged 79-87 years) during 2016-2017. Meanwhile, the US Health, Aging, and Body Composition (HABC) Study included both male and female participants (n=2998, aged 71-77 years) in 1998-1999."

Discussion:

The discussion would benefit from a more critical analysis concerning the observed association. It currently overlooks the general deterioration in physiological systems associated with aging, which might explain these findings. Additionally, the possibility of unmeasured confounders has not been adequately considered.

**Reviewer**	**2**
**Name**	**Chan, Karina**
**Affiliation**	**University of New South Wales, Centre for Healthy Brain Ageing**
**Date**	**11-Jul-2024**
**COI**	**Nil.**

Thank you for the opportunity to review this manuscript. This is an interesting study examining the relationship between various indicators of oral health and appetite decline in older adults. Research examining factors which may contribute to healthy ageing is particularly important given rising life expectancy and population aging. The prospective nature of this cohort study builds upon prior research and strengthens the validity of the study's findings.

My main comments for revision are as follows. First, given the complexity of the two cohort studies, a clearer description of the methods section is necessary. A figure or table may be helpful for clearly displaying the measures used in both cohorts across the two timepoints. Second, while the paper makes reference to both objective and subjective measures of oral health, the majority of measures and analyses focus on subjective reports. The implications of this should be considered in the discussion. Third, the discussion would benefit from greater exploration of mechanisms by which oral health may lead to deterioration in appetite (e.g. changes in taste, food preference, and swallowing difficulties). Fourth, the discussion would benefit from greater consideration of the practical implications of the findings, in addition to policy makers addressing barriers to maintain good oral health. I have provided an attachment with more specific comments and suggestions. Overall, this is a promising study that could make a valuable contribution to the field with revisions.

- Page 4 Line 18-28: First, please consider citing a more recent source given much research in the area since the cited source. Second, please review accuracy of information. Social factors such as poverty, difficulty in shopping and preparing food, lack of socialisation, and inability to feed oneself may have greater contributions to weight loss than appetite loss, which is the focus of this study. Dementia is not considered a psychological factor, and would be more appropriately conceptualised as a neurocognitive factor or something similar. Fourth, taste, vision, and smell, are not hedonic qualities – they are senses. Stimuli which are perceived by these senses, may have hedonic qualities (e.g., smell of bacon, sight of pink icing).
- Page 5 line 55: The response rates between the studies are very different, and a 99+% response rate for HABC is quite extraordinary. Is there an explanation for why the response rates were so high in HABC?
- Page 6: Consider providing a figure or table to make clear which measures of oral health were available at baseline and follow-up for each study, as this section was difficult to follow. For example, page 5 Line 55/Page 6 Line 1 states that participants were followed up for physical assessment and questionnaires. Given the description of physical

assessment/questionnaires provided for baseline, this leads the reader to believe oral health measures were included. The *measures of oral health* section also do not clarify this, and it is not apparent that follow-up assessment for HABC cohort did not include measures of oral health until Page 6 Line 44-46.

- Page 6 Line 13: Please indicate response options for number of natural teeth in BRHS study. If response option is categorical (yes/no) then *presence* of natural teeth may be the more appropriate descriptor of this question.
- Page 6 Line 26: Please specify response options for difficulty eating or chewing foods due to dental problem and food avoidance due to dental problems or change grammatical structure of sentence if all of these questions used yes/no response options similar to denture/s use.
- Page 6 Line 56: Please reference the use of > 14 as a cut-off for the SNAQ.
- Page 7 Line 2-6: Please clarify whether information on *SNAQ score collected at follow-up* was used to determine change in appetite loss over time, or whether the *difference in SNAQ scores from baseline to follow-up* were used to determine change in appetite over time? If the former, please justify why baseline data was not considered. If the latter, please edit. This will have implications for the results.
- Page 7 Line 16: Please explain why 'moderate' appetite is categorised as appetite loss? Please also explain how changes in appetite loss overtime variable was calculated.
- Page 7 line 32: Consider whether social class categories should be provided.
- Page 7 Line 35: If possible, consider providing number of years since smoking cessation as opposed to specific dates to improve readability.
- Page 7: Inclusion criteria for history of CD for BRHS is provided but not for HABC. Consider including.
- Page 8 Line 30. Specify that cross-sectional relationships being explored refer to baseline data of these studies.
- Page 9: Text states 53% of HABC study is female which is at odds with the 52% of males reported in Table 1 page 16.
- Page 9 22: Comparing manual occupational social class to high school graduation attainment is an unusual demographic comparison when more equivalent comparisons of educational attainment can be made from the descriptive data available. Please revise.
- Page 9 Line 32: Specify that cross-sectional relationships being explored refer to baseline data of these studies.
- Page 9 Line 37: Please consider reporting the fully adjusted model only or justify why age only adjusted model has been presented in some cases whereas fully adjusted model has been presented in others. Same for Page 10/Table 3.
- Page 9 Line 44-50: OR and relevant descriptive statistics for those who had loose dentures and teeth sensitivity is not provided in Table 2. Please include.
- Page 9 Line 55: Specify that findings are true of both models.
- Page 9 and 10 general comments: Consider providing some consistency as to whether ORs are reported in text given that they are already in table. It is not clear why some significant ORs have been placed in text while others have only been placed in table.
- Page 10: Please consider including significant OR of >1 cumulative oral health problem. Please also consider including that Denture use was significant in age-adjusted but not fully adjusted model.
- Page 10 Line 16: Please specify that analyses is regarding oral health measures *at baseline*
- Page 12 Line 36: "…thereby maintaining adequate appetite etc." is too strong of a statement to make from correlational data. Please review.

- Page 16: 'Other' is not the most appropriate category for HABC study Race when it entirely comprises African Americans – please amend either by changing descriptor name or adding note to table.
- Page 16: Levels of smoking status accurately describe the HABC study but not BRHS study, per information provided on page 7. Please revise.
- Page 17: Consider including range, M, and SD for cumulative measure of oral health (either in table or in text with results).
- Page 17. Please consider changing 'food avoidance' to 'food avoidance due to dental problems' for clarity, given outcome measure is appetite loss.

General comments –

- Since what is defined as baseline and follow-up in the current study is clearly described on page 6, I suggest you use that terminology throughout to optimise readability (reference to 2016-2017, Year 2 etc. makes it less clear).
- Please re-word to specify what is meant by *socially representative sample* written on Page 5 and in the discussion, given that lack of generalisability from limited sample demographics was indicated as a limitation.

VERSION 1 - AUTHOR RESPONSE

Reviewer: 1 Dr. Faisal Hakeem, King's College London

Comments to the Author: This study presents a compelling analysis of the cross-sectional and longitudinal relationship between oral health and appetite loss among two cohorts of older adults. The research contributes valuable insights to the existing literature. Despite several limitations, the authors have adeptly addressed these in the discussion section. Below are a few minor suggestions aimed at improving the manuscript's readability.

1. Abstract: Please specify the age range for clarity: "The British Regional Heart Study (BRHS) encompassed male 0 (n=1348, aged 79-87 years) during 2016-2017. Meanwhile, the US Health, Aging, and Body Composition (HABC) Study included both male and female participants (n=2998, aged 71-77 years) in 1998-1999."

Authors' response: Thank you for this comment. The sample population in the BRHS study was aged 79-87 at baseline and was followed up after 2 years, when they were aged 81-89 years. Similarly, the sample population in the HABC Study was 71-77 years at baseline and was followed up after 2 years, when they were aged 73-79 years. We have clarified this in the abstract now.

Changes made to the manuscript: As suggested by the Reviewer, we have amended the description about the study participants in the Abstract to specify their age range at baseline and at follow-up to provide more clarity.

2. Discussion: The discussion would benefit from a more critical analysis concerning the observed association. It currently overlooks the general deterioration in physiological

systems associated with aging, which might explain these findings. Additionally, the possibility of unmeasured confounders has not been adequately considered.

Authors' response: Thank you for this comment. We have now expanded the discussion section to provide a more detailed analysis concerning the observed associations. In particular, we agree with the suggestion from the Reviewer of the possibility that the association could be due to general age-related deterioration in physiological systems. We also agree that the possibility of residual confounding remains due to unmeasured confounders such as dental history, reasons for tooth loss, and inadequately capturing cofounders such as socioeconomic factors and behavioural factors and their influence across the lifecourse (including diet, smoking), and co-morbidities such as dementia and cognitive decline. We have addressed these additional points in the Discussion section.

Changes made to the manuscript: As suggested, we have elaborated further in the Discussion to provide a more critical analysis of the findings observed, including aspects of residual confounding, and age-related decline in physiological systems (page 13 paragraph 2).

Reviewer: 2 Ms. Karina Chan, University of New South Wales

Comments to the Author: Thank you for the opportunity to review this manuscript. This is an interesting study examining the relationship between various indicators of oral health and appetite decline in older adults. Research examining factors which may contribute to healthy ageing is particularly important given rising life expectancy and population aging. The prospective nature of this cohort study builds upon prior research and strengthens the validity of the study's findings.

My main comments for revision are as follows.

1. First, given the complexity of the two cohort studies, a clearer description of the methods section is necessary. A figure or table may be helpful for clearly displaying the measures used in both cohorts across the two timepoints.

   Authors' response: Thank you for this comment. We agree that it would be beneficial to include a table showing different measures of oral health available at baseline and at follow-up in the two cohort studies.
   Changes made to the manuscript: As suggest by the Reviewer, we have added Appendix 1 Table 1 to present the key oral health measures available at baseline and follow-up in the BRHS and HABC Study.

2. Second, while the paper makes reference to both objective and subjective measures of oral health, the majority of measures and analyses focus on subjective reports. The implications of this should be considered in the discussion.
   Authors' response: Thank you for this comment. We recognise that most oral health measures in both studies were of subjective nature and the impact of these measures on the observed findings needs discussion. Also, the need for investigating the role of

objective measures of oral health such as number of teeth, bleeding gums on appetite loss needs to be examined in further research.
Changes made to the manuscript: We have amended the Discussion section to specify the subjective nature of the most oral health measures available in both cohorts and need for future exploration to study the impact of objective oral health measures on appetite in older age. (page 13 paragraph 1)

3. Third, the discussion would benefit from greater exploration of mechanisms by which oral health may lead to deterioration in appetite (e.g. changes in taste, food preference, and swallowing difficulties).

Authors' response: Thank you for this comment. We agree that it would be beneficial to have greater detail about the mechanisms by which oral health measures could influence appetite loss. We acknowledge that age-related decline in health, particularly changes in taste, food preference, and swallowing difficulties could be relevant mechanisms to add to the Discussion. .
Changes made to the manuscript: We have added to the discussion section to elaborate on the the mechanisms by which oral health may lead to deterioration in appetite in later life (page 12 paragraph 2).

4. Fourth, the discussion would benefit from greater consideration of the practical implications of the findings, in addition to policy makers addressing barriers to maintain good oral health.

Authors' response: Thank you for this comment. We acknowledge that policy implications of the findings could be further strengthened. As the Reviewer suggests, there are important implications for policy makers to consider for addressing barriers to maintaining good oral health in older age – this includes action and policy implications at different levels, including both early prevention across the lifecourse, as well as adequate access to dental services, and promoting oral health awareness and including opportunistic dental health promotion for older adults embedded in other health screening activities.
Changes made to the manuscript: We have amended the discussion section to include the practical implications of this study. (page 13 last paragraph)

Additional Comments from Reviewer 2

I have provided an attachment with more specific comments and suggestions. Overall, this is a promising study that could make a valuable contribution to the field with revisions.

5. Page 4 Line 18-28: First, please consider citing a more recent source given much research in the area since the cited source. Second, please review accuracy of information. Social factors such as poverty, difficulty in shopping and preparing food, lack of socialisation, and inability to feed oneself may have greater contributions to weight loss than appetite loss, which is the focus of this study. Dementia is not considered a psychological factor, and would be more appropriately conceptualised as a neurocognitive factor or something similar. Fourth, taste, vision, and smell, are not hedonic qualities – they are senses.

Stimuli which are perceived by these senses, may have hedonic qualities (e.g., smell of bacon, sight of pink icing).

Authors' response: Thank you for this comment.

-We recognise that the references used to describe the factors associated with appetite loss in older age need to be updated to add more recent sources.

-We understand that the research suggests that the social factors described may have greater contribution to weight loss, however, the mechanisms of actions of these associations remain underexplored and some studies suggests that these factors may influence the appetite in old age.

-We agree that dementia could be more appropriately considered as a neurocognitive factor. We recognise that the term 'sensory impairment' would be more appropriate in this context to describe the decline in aspects of hearing, smell and taste, which are more common in old age and may affect appetite.

Changes made to the manuscript:

As suggested by the Reviewer, we have added more recent references in the Introduction section related to appetite loss in older age and associated factors.

-We have replaced the term 'hedonic qualities' with a more appropriate term 'sensory impairment' alongside other changes suggested by the reviewer. (page 4 paragraph 1)

6. Page 5 line 55: The response rates between the studies are very different, and a 99+% response rate for HABC is quite extraordinary. Is there an explanation for why the response rates were so high in HABC?
   Authors' response: Thank you for this comment. In the HABC Study, all participants were free of disability and able to walk a quarter of a mile or climb 10 steps without a difficulty at the time of initial examination. Additionally, the data collection was done through both clinic and home visits, which may have resulted in the higher response rate.
   Changes made to the manuscript: We have an explanation on this point in the Methods section of the revised manuscript. (Page 5 last paragraph)

7. Page 6: Consider providing a figure or table to make clear which measures of oral health were available at baseline and follow-up for each study, as this section was difficult to follow. For example, page 5 Line 55/Page 6 Line 1 states that participants were followed up for physical assessment and questionnaires. Given the description of physical assessment/questionnaires provided for baseline, this leads the reader to believe oral health measures were included. The measures of oral health section also do not clarify this, and it is not apparent that follow-up assessment for HABC cohort did not include measures of oral health until Page 6 Line 44-46.
   Authors' response: Thank you for this comment. We agree that it will be beneficial to provide a table to show different measures of oral health available at baseline and at follow-up in the BRHS.
   Changes made to the manuscript: We have now added Appendix 1 Table 1 to present the key oral health measures available at baseline and follow-up in the BRHS and HABC Study.

8. Page 6 Line 13: Please indicate response options for number of natural teeth in BRHS study. If response option is categorical (yes/no) then presence of natural teeth may be the more appropriate descriptor of this question.
   Authors' response: Thank you for this comment. Dental data were collected at baseline (2016-17) and at follow up (2018-19) in the BRHS. At baseline, only one question (Do you have any of your natural teeth?) was asked to the participants. At follow-up, in addition to asking this question within the questionnaires, oral health exams were carried out by dental professionals who counted the number of natural teeth present in participants. Therefore, the number of teeth was a continuous variable which could provide range from 0 to 32 teeth. We have amended the description to add more clarity for the readers. However, a continuous measure of tooth count was not available at baseline and therefore, it was recoded into a categorical variable of tooth loss (categories: whether or not have natural teeth) to aid the longitudinal analysis. Similarly, we recoded this variable in the HABC study as well to make the data available in both BRHS and HABC studies comparable. This is mentioned under the 'statistical analysis section' as follows- For oral health measures, tooth loss was categorized as with or without any natural teeth (i.e., complete tooth loss) in the BRHS and the HABC Study.

   Changes made to the manuscript: We have added further description to the responses for the variable on the number of natural teeth in the BRHS study. (Page 6 paragraph 2)

9. Page 6 Line 26: Please specify response options for difficulty eating or chewing foods due to dental problem and food avoidance due to dental problems or change grammatical structure of sentence if all of these questions used yes/no response options similar to denture/s use.
   Authors' response: Thank you for this comment. We agree to provide the categories of above-mentioned variables.
   Changes made to the manuscript: We have now described the categories of the variables on difficulty eating or chewing foods due to dental problem and food avoidance due to dental problems in the Methods section of the manuscript (page 6 paragraph 3).

10. Page 6 Line 56: Please reference the use of > 14 as a cut-off for the SNAQ.
    Authors' response: Thank you for this comment. We agree that the cut-off used needs to be clarified in this section.
    Changes made to the manuscript: We have added the reference for using > 14 as a cut-off for the SNAQ. (page 7 paragraph 1)

11. Page 7 Line 2-6: Please clarify whether information on SNAQ score collected at follow-up was used to determine change in appetite loss over time, or whether the difference in SNAQ scores from baseline to follow-up were used to determine change in appetite over time? If the former, please justify why baseline data was not considered. If the latter, please edit. This will have implications for the results.
    Authors' response: Thank you for this comment. We examined the impact of poor oral health on appetite loss through two approaches. In the first approach, we examined cross-sectional relationships between measures of poor oral health and appetite loss at baseline.

For this, we used the SNAQ scores from baseline. In the second approach, we examined the impact of poor oral health on changes in appetite loss over time. For this, we used SNAQ scores from baseline and follow-up to determine changes in appetite loss over time.

Changes made to the manuscript: We have revised the Methods section of the manuscript to provide further clarification on the information used to assess associations presented in the findings (page 7 paragraph 1).

12. Page 7 Line 16: Please explain why 'moderate' appetite is categorised as appetite loss? Please also explain how changes in appetite loss overtime variable was calculated.
Authors' response: Thank you for this comment. In year 2 of the HABC Study, appetite loss was determined through a self-reported question and the participants reporting having moderate/poor/very poor appetite were categorised as having appetite loss. This was the grouping used in a previous paper in the HABC study on appetite loss [Lee JS, Kritchevsky SB, Tylavsky F, et al. Factors Associated with Impaired Appetite in Well-Functioning Community-Dwelling Older Adults. Journal of Nutrition For the Elderly 2006;26(1-2):27-43. doi: 10.1300/J052v26n01_02]. This reference is cited in the manuscript. We also agree that more clarity on how changes in appetite loss overtime is needed in the manuscript.

Changes made to the manuscript: We have revised the manuscript (Methods section) to state that in both studies, changes in appetite loss were categorised as sustained good/improved (no appetite loss at baseline and at follow-up; or appetite loss at baseline but not at follow-up) and sustained poor/deteriorated (appetite loss at baseline and at follow-up; or no appetite loss at baseline but reported appetite loss at follow-up) (page 7 paragraph 3).

13. Page 7 line 32: Consider whether social class categories should be provided.
Authors' response: Thank you for this comment. We agree that the categories/ groupings of occupational social class would be useful to add. Occupational social class categories were based on the Registrar General's Social Class classification. This was determined by the longest-held occupation recorded in the BRHS at study entry (aged 40–59 years) and comprised six social class groups— I (professionals, eg, physicians, engineers), II (managerial, e.g., teachers, sales managers), III non-manual (semiskilled non-manual, e.g., clerks, shop assistants), III manual (semiskilled manual, e.g., bricklayers), IV (partly skilled, e.g., postmen) and V (unskilled, e.g., porters, general labourers). For the purposes of this study, social classes III manual, IV and V were grouped as manual social class. This was in line with the previous study undertaken using the BRHS data [Ramsay SE, Whincup PH, Watt RG, et al. Burden of poor oral health in older age: findings from a population-based study of older British men. *BMJ Open* 2015;5(12):e009476. doi: 10.1136/bmjopen-2015-009476].

Changes made to the manuscript: We have amended the manuscript to describe the categories of social class (based on the Registrar General's Social class Classification),

and have added a reference to the above-mentioned study which had employed similar categories of social class (page 7 paragraph 4).

14. Page 7 Line 35: If possible, consider providing number of years since smoking cessation as opposed to specific dates to improve readability.
Authors' response: Thank you for this comment. The BRHS involved a series of questionnaire surveys (annual data collection) which enquired about whether or not the participants were smoking at the time of completing the survey and if they had any history of smoking. Thus, the variables used in the study were recoded to the year of the survey data collection and involved three categories- never smoked, a current smoker, or a former smoker. We have corrected these in the manuscript.

    Changes made to the manuscript: The categories for smoking status are now specified in the manuscript (Page 7 paragraph 4).

15. Page 7: Inclusion criteria for history of CD for BRHS is provided but not for HABC. Consider including.
Authors' response: Thank you for this comment. History of cardiovascular disease in the HABC Study was based on self-reported doctor-diagnosis of heart attack, angina, stroke, or heart failure.
Changes made to the manuscript: As suggested by the Reviewer, we have edited the Methods section to clarify the inclusion criteria for the history of cardiovascular disease in the HABC Study (page 8 paragraph 2).

16. Page 8 Line 30. Specify that cross-sectional relationships being explored refer to baseline data of these studies.
Authors' response: Thank you for this comment. We agree that specifying baseline data being used to assess cross-sectional relationships would provide more clarity for the readers.

    Changes made to the manuscript: We have revised the manuscript to state that logistic regression was undertaken to examine cross-sectional relationships between each oral health marker and appetite loss, separately using the baseline data from both studies, to obtain odds ratios (ORs) and 95% confidence intervals (95%CIs) (page 8, last paragraph).

17. Page 9: Text states 53% of HABC study is female which is at odds with the 52% of males reported in Table 1 page 16.
Authors' response: Thank you for this comment and for pointing out this typo. We have corrected the statement in the manuscript in line with Table 1.
Changes made to the manuscript: We have corrected the information in the manuscript where we state that 52% of participants in the HABC Study were male (page 9 paragraph 4).

18. Page 9 22: Comparing manual occupational social class to high school graduation attainment is an unusual demographic comparison when more equivalent comparisons of educational attainment can be made from the descriptive data available. Please revise.
    Authors' response: Thank you for this comment. We have amended the statements in question to reflect that we are not comparing occupational social class to high school graduation attainment.
    Changes made to the manuscript: We have revised the manuscript to have two separate statements stating that 45% participants were from manual occupational social class in the BRHS and 24% participants did not attain high school graduation in the HABC Study (page 9, paragraph 4).

19. Page 9 Line 32: Specify that cross-sectional relationships being explored refer to baseline data of these studies.
    Authors' response: Thank you for this comment. We agree that specifying baseline data being used to assess cross-sectional relationships would provide more clarity for the readers.

    Changes made to the manuscript: We have revised the first line of the last paragraph of page 10 to specify that Table 2 presents ORs (95%CIs) for appetite loss according to measures of oral health from cross-sectional analyses undertaken using baseline data from the BRHS and the HABC Study (page 10, paragraph 1).

20. Page 9 Line 37: Please consider reporting the fully adjusted model only or justify why age only adjusted model has been presented in some cases whereas fully adjusted model has been presented in others. Same for Page 10/Table 3.

    Authors' response: Thank you for this comment. In cross-sectional analyses in the BRHS, we observed that 'teeth sensitivity' was not significantly associated with 'appetite loss' even when only adjusting for 'age'. Therefore, we have reported this in the Results section of the manuscript. While reporting the results from Table 3, we have stated that - Participants with >2 dry mouth symptoms had a greater risk of poor/deterioration in appetite [age-adjusted OR=2.30 (95%CI=1.56-3.38)] compared to those without dry mouth symptoms, and this remained significant on full adjustment. Here, we want to highlight that we observed significant association between dry mouth symptoms and appetite loss in both age-adjusted and fully-adjusted models.

21. Page 9 Line 44-50: OR and relevant descriptive statistics for those who had loose dentures and teeth sensitivity is not provided in Table 2. Please include.
    Authors' response: Thank you for this comment. The variables on loose dentures and teeth sensitivity were only available in the BRHS study and not in the HABC Study. Therefore, we have reported these in the Results section of the manuscript. Table 2 provides the results for variables which were available in both studies, to help the readers see these together and to compare the findings from the two studies.

22. Page 9 Line 55: Specify that findings are true of both models.

Authors' response: Thank you for this comment. We agree and recognise the importance of stating in the manuscript that the findings relating to HABC Study from Table 2 were true for both models of adjustment.
Changes made to the manuscript: As suggested by the Reviewer, we have revised the statement in the manuscript to specify that the risk of appetite loss was greater in participants with poor self-rated oral health, dry mouth, having difficulty eating or chewing foods, avoiding foods, and those with having ≥2 oral health problems, which was observed in both age-adjusted and fully adjusted models (page 10 paragraph 1).

23. Page 9 and 10 general comments: Consider providing some consistency as to whether ORs are reported in text given that they are already in table. It is not clear why some significant ORs have been placed in text while others have only been placed in table.
Authors' response: Thank you for this comment. Due to the number of oral health measures being used in both cohorts, we were unable to provide all odds ratios (ORs) in the text of the Results section as it would increase the word count and to avoid unnecessary duplication. However, we have presented a few significant ORs in the text to highlight the patterns observed across these cohorts. In Table 2, the ORs are presented for the oral health measures that were available for both cohorts to enable comparison of the findings from both cohorts. Additionally, we have provided the ORs in the text where the oral health measures were available in only one cohort study.

24. Page 10: Please consider including significant OR of >1 cumulative oral health problem. Please also consider including that Denture use was significant in age-adjusted but not fully adjusted model.
Authors' response: Thank you for this comment. Assuming that the Reviewer is referring to the description of findings from Table 3 presented in the text, we agree to provide the significant OR of >1 cumulative oral health problem and also briefly describe that the denture use was significant in age-adjusted but not fully adjusted model.
Changes made to the manuscript: As suggested, we have amended the Results section to add the significant OR of >1 cumulative oral health problem and briefly describe that the denture use was significant in age-adjusted but not fully adjusted model (page 10 paragraph 2).

25. Page 10 Line 16: Please specify that analyses is regarding oral health measures at baseline
Authors' response: Thank you for this comment. We have made this correction.
Changes made to the manuscript: We have amended the manuscript to describe that Table 4 presents results from prospective analyses between oral health measures at baseline and changes in appetite loss from baseline to follow-up in the HABC Study. (page 11 paragraph 1)

26. Page 12 Line 36: "…thereby maintaining adequate appetite etc." is too strong of a statement to make from correlational data. Please review.

Authors' response: Thank you for this comment. We agree that the statement "…thereby maintaining adequate appetite etc." may suggest a causal relationship between poor oral health and loss of appetite and we agree to revise it.

Changes made to the manuscript: We have revised this statement to suggest that addressing barriers to maintaining good oral health may facilitate maintaining adequate appetite. (page 13 paragraph 3)

27. Page 16: 'Other' is not the most appropriate category for HABC study Race when it entirely comprises African Americans – please amend either by changing descriptor name or adding note to table.

    Authors' response: Thank you for this comment. We agree that clarification is needed to describe the category 'Other' for the variable 'Race' in the HABC Study, which was entirely comprised of African American adult population.

    Changes made to the manuscript: As suggested, we have added a note specifying the 'Other' category of Race for the HABC Study in Table 1 (page 18).

28. Page 16: Levels of smoking status accurately describe the HABC study but not BRHS study, per information provided on page 7. Please revise.

    Authors' response: Thank you for this comment. We have revised the sentence on page 7 describing the categories of smoking status in the BRHS in line with the categories mentioned in Table 1 that were used for the analysis.

    Changes made to the manuscript: We have revised the description of categories for smoking status in the BRHS as current smokers, former smokers, and those who never smoked. (page 7 paragraph 4).

29. Page 17: Consider including range, M, and SD for cumulative measure of oral health (either in table or in text with results).

    Authors' response: Thank you for this suggestion. We agree that it would be useful to include an overall descriptive statistics for cumulative measure of oral health. Since this is an ordinal variable (three categories of 0, 1 and 2), we have included the percentage of these categories in Table 2. We have also included the percentages for all oral health measures to ensure consistency.

    Changes made to the manuscript: We have now included the percentages for cumulative oral health measure and for all oral health measures for both studies in Table 2. (page 19)

30. Page 17. Please consider changing 'food avoidance' to 'food avoidance due to dental problems' for clarity, given outcome measure is appetite loss.

    Authors' response: Thank you for this comment. We agree that the variable could be more accurately described as 'food avoidance due to dental problems'.

    Changes made to the manuscript: We have amended the name of the variable on food avoidance due to dental problems in Tables 2 to 5 (page 19 to 22).

31. **General comments –**
    Since what is defined as baseline and follow-up in the current study is clearly described on page 6, I suggest you use that terminology throughout to optimise readability (reference to 2016-2017, Year 2 etc. makes it less clear).
    Authors' response: Thank you for this comment. We understand the necessity of using consistent terminology for describing the baseline and follow-up time periods throughout the manuscript.
    Changes made to the manuscript: We have amended the manuscript to ensure the use of consistent terminologies when referring to the baseline and follow-up.

32. Please re-word to specify what is meant by socially representative sample written on Page 5 and in the discussion, given that lack of generalisability from limited sample demographics was indicated as a limitation
    Authors' response: Thank you for this comment. The term 'socially representative sample' for the BRHS study referred to the socioeconomic and geographic representativeness of the study population. This was based on the sample being broadly representative of the general populations in terms of social class (participants longest held occupation) and of all major geographic regions in the country. However, a limitation of the sample demographics is its generalisability to women and non-White ethnic groups. We acknowledge that this point needs clarification in the manuscript.

    Changes made to the manuscript: We have amended the Methods section of the manuscript to specify that 'socially representative sample' referred to socioeconomic and geographic representativeness of the sample (page 5 paragraph 2 and 3).

VERSION 2 - REVIEW

**Reviewer**	**2**
**Name**	**Chan, Karina**
**Affiliation**	**University of New South Wales, Centre for Healthy Brain Ageing**
**Date**	**08-Jan-2025**
**COI**	

Thank you for the opportunity to review this manuscript again and for addressing the concerns raised previously. This is an interesting study that makes a valuable contribution to the literature in this area.
